# Fast and Accurate Multivariate Gaussian Modeling of Protein Families: Predicting Residue Contacts and Protein-Interaction Partners

**DOI:** 10.1371/journal.pone.0092721

**Published:** 2014-03-24

**Authors:** Carlo Baldassi, Marco Zamparo, Christoph Feinauer, Andrea Procaccini, Riccardo Zecchina, Martin Weigt, Andrea Pagnani

**Affiliations:** 1 Department of Applied Science and Technology and Center for Computational Sciences, Politecnico di Torino, Torino, Italy; 2 Human Genetics Foundation-Torino, Torino, Italy; 3 Sorbonne Universités, Université Pierre et Marie Curie Paris 06, UMR 7238, Computational and Quantitative Biology, Paris, France; 4 Centre National de la Recherche Scientifique, UMR 7238, Computational and Quantitative Biology, Paris, France; Technical University Darmstadt, Germany

## Abstract

In the course of evolution, proteins show a remarkable conservation of their three-dimensional structure and their biological function, leading to strong evolutionary constraints on the sequence variability between homologous proteins. Our method aims at extracting such constraints from rapidly accumulating sequence data, and thereby at inferring protein structure and function from sequence information alone. Recently, global statistical inference methods (e.g. direct-coupling analysis, sparse inverse covariance estimation) have achieved a breakthrough towards this aim, and their predictions have been successfully implemented into tertiary and quaternary protein structure prediction methods. However, due to the discrete nature of the underlying variable (amino-acids), exact inference requires exponential time in the protein length, and efficient approximations are needed for practical applicability. Here we propose a very efficient multivariate Gaussian modeling approach as a variant of direct-coupling analysis: the discrete amino-acid variables are replaced by continuous Gaussian random variables. The resulting statistical inference problem is efficiently and exactly solvable. We show that the quality of inference is comparable or superior to the one achieved by mean-field approximations to inference with discrete variables, as done by direct-coupling analysis. This is true for (*i*) the prediction of residue-residue contacts in proteins, and (*ii*) the identification of protein-protein interaction partner in bacterial signal transduction. An implementation of our multivariate Gaussian approach is available at the website http://areeweb.polito.it/ricerca/cmp/code.

## Introduction

One of the most important challenges in modern computational biology is to exploit the wealth of sequence data, accumulating thanks to modern sequencing technology, to extract information and to reach an understanding of complex biological processes. A particular example is the inference of conserved structural and functional properties of proteins from the empirically observed variability of amino-acid sequences in homologous protein families, e.g. via the inference of signals of co-evolution between residues, which may be distant along the sequence, but in contact in the folded protein; cf. [1]–[6] for a selection of classical works and [7] for a review over recent developments. In the last 5 years, a strong renewed interest in residue co-evolution has been emerging: a number of global statistical inference approaches [8]–[16] have led to a highly increased precision in predicting residue contacts from sequence information alone. Furthermore, co-evolutionary analysis was found to provide valuable insight on specificity and partner prediction in protein-protein interaction [17], [18] in bacterial signal transduction.

Key to this recent progress are *global statistical inference* approaches, like *direct-coupling analysis* (DCA) [8], [10] and *sparse inverse covariance estimation* (PSICOV) [12], and the GREMLIN algorithm based on *pseudo-likelihood maximization* [11], [16]. DCA is based on the maximum-entropy (MaxEnt) principle [19], [20] which naturally leads to statistical models of protein families in terms of so-called Potts models or Markov random fields. Proposed initially more than a decade ago [21], [22], it was not until very recently that the first successful MaxEnt approaches to the study of co-evolution were published [8], [23]. The main idea behind such global inference techniques is the following: correlations between the amino-acids occurring in two positions in a protein family, i.e. between two columns in the corresponding multiple-sequence alignment (MSA), may result not only from direct co-evolutionary couplings. They may also be generated by a whole network of such couplings. More precisely, if a position 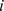 is coupled to a position 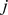, and 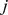 is coupled to 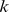, then 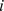 and 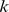 will also show some correlation even if they are not coupled. The aim of global methods is to disentangle such direct and indirect effects, and to infer the network of direct co-evolutionary couplings starting from the empirically observed correlations.

In this context, we focus on two different biological problems: the inference of residue-residue contacts and the prediction of interaction partners.

The inference of residue-residue contacts from large MSAs of homologous proteins [8]–[16] is an important challenge in structural biology. Inferred contacts have been shown to be sufficient to guide the assembly of complexes between proteins of known (or homology modeled) monomer structure [24], [25], and to predict the fold of single proteins [26]–[31], including highlights like large trans-membrane proteins [28], [31]. In [25], the predicted structure of the auto-phosphorylation complex of a bacterial histidine sensor kinase has been used to repair a non-functional chimeric protein by rationally designed mutagenesis; this structure is also, to the best of our knowledge, the first case of a prediction, which has subsequently been confirmed by experimental X-ray structures [32], [33]. The possibility to guide tertiary and quaternary protein structure prediction is an important finding, in light of the experimental effort needed for generating high-resolution structures.

The second problem, concerning molecular determinants of interaction specificity of proteins and the identification of interaction partners [17], [18], is a central problem in systems biology. In both cited papers, bacterial two-component signal transduction systems (TCS) were chosen, which constitute a major way by which bacteria sense their environment, and react to it [34]. TCS consist of two proteins, a histidine sensor kinase (SK) and a response regulator protein (RR): the SK senses an extracellular signal, and activates a RR by phosphorylation; the RR typically acts as a transcription factor, thus triggering a transcriptional response to the external signal. The same (homologous) phosphotransfer mechanism is used for several signaling pathways in each bacterium; thus, to produce the correct cellular response to an external signal, interactions have to be highly specific inside each pathway: crosstalk between pathways has to be avoided [35]–[37]. This evolutionary pressure can be detected by co-evolutionary analysis [17], [18]. Results are interesting: statistical couplings inferred by DCA reflect physical interaction mechanisms, with the strongest signal coming from charged amino-acids. They are able to predict interacting SK/RR pairs for so-called orphan proteins (SK and RR proteins without an obvious interaction partner), and the predictions compared favorably to most available experimental results, including the prediction of 7 (out of 8 known) interaction partners of orphan signaling proteins in *Caulobacter crescentus* [18].

In the present study, we describe an alternative approach to co-evolutionary analysis, based on a multivariate Gaussian modeling of the underlying MSA. It can be understood as an approximation to the MaxEnt Potts model in which (*i*) the discreteness constraint is released, i.e. continuous values are allowed for variables representing amino-acids, (*ii*) a Gaussian interaction model is assumed, and (*iii*) a prior distribution is introduced to compensate for the under-sampling of the data. This simplification allows to explicitly determine the model parameters from empirically observed residue correlations. The approach shares many similarities with [12], in which a multivariate Gaussian model is also assumed, and with the mean-field approximation to the discrete DCA model [10], but the simpler structure of the probability distribution makes the model analytically tractable, and allows for an efficient implementation, while still having a prediction accuracy comparable or superior to that of the aforementioned models (see the Results section). The model is briefly described in the next section, and in greater detail in the Materials and Methods section.

A fast, parallel implementation of the multivariate Gaussian modeling approach is provided on http://areeweb.polito.it/ricerca/cmp/code in two different versions, a MATLAB [38] one and a Julia [39] one.

### Gaussian Modeling of Multiple Sequence Alignments

This section briefly outlines the prediction procedure coming from our proposed model, and highlights its main distinctive features with respect to other similar methods. A full presentation can be found in the Materials and Methods section, and additional details in File S1.

The input data to our model is the MSA for a large protein-domain family, consisting of 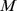 aligned homologous protein sequences of length 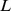. Sequence alignments are formed by the 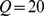 different amino-acids, and may contain alignment gaps.

As in [12], we consider a multivariate Gaussian model in which each variable represents one of the 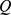 possible amino-acids at a given site, and aim in principle at maximizing the likelihood of the resulting probability distribution given the empirically observed data (in particular, given the observed mean and correlation values, computed according to a reweighting procedure devised to compensate for the sampling bias). Doing so would yield the parameters for the most probable model which produced the observed data, which in turn would provide a synthetic description of the underlying statistical properties of the protein family under investigation. Unfortunately, however, this is typically infeasible, due to under-sampling of the sequence space. A possible approach to overcome this problem, used e.g. in [12], is to introduce a sparsity constraint, in order to reduce the number of degrees of freedom of the model. Here, instead, we propose a Bayesian approach, in which a suitable prior is introduced, and the parameter estimation is then performed over the posterior distribution.

A convenient choice for the prior is the normal-inverse-Wishart (NIW), which, being the conjugate prior of the multivariate Gaussian distribution, provides a NIW posterior. Thus, within this choice, the posterior simply is a data-dependent re-parametrization of the prior: as a result, the problem is analytically tractable, and the computation of relevant quantities can be implemented efficiently. Furthermore, by choosing the parameters for the prior to be as uninformative as possible (i.e. corresponding to uniformly distributed samples), we obtain an expression for the posterior which, interestingly, can be reconciled with the pseudo-count correction of [10]: in the Gaussian framework, the pseudo-count parameter has a natural interpretation as the weight attributed to the prior.

We then estimate the parameters of the model as averages on the posterior distribution, which have a simple analytical expression and can be computed efficiently (in practical terms, the computation amounts to the inversion of a 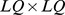 matrix). On one hand, this yields an estimate of the strengths of direct interactions between the residues of the alignments, which can be used to predict protein contacts. On the other hand, this allows to build joint models of interacting proteins, which can be used to score candidate interaction partners, simply by computing their likelihood - which can be done very efficiently on a Gaussian model.

The contact prediction between residues relies on the model's inferred interaction strengths (i.e. couplings), which are represented by 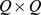 matrices; in order to rank all possible interactions, we need to compute a single score out of each such matrix. As mentioned above, these matrices are numerically identical to those obtained in the mean-field approximation of the discrete (Potts) DCA model. We tested two scoring methods: the so-called direct information (DI), introduced in [8], and the Frobenius norm (FN) as computed in [15]. The DI is a measure of the mutual information induced only by the direct couplings, and its expression is model-dependent: in the Gaussian framework it can be computed analytically (see File S1) and yields slightly different results with respect to the Potts model (but with a comparable prediction power, see the Results section). The FN, on the other hand, does not depend on the model, and therefore some of the results which we report here for the contact prediction problem are applicable in the context of the Potts model as well. In our tests, the FN score yielded better results; however, the DI score is gauge-invariant and has a well-defined physical interpretation, and is therefore relevant as a way to assess the predictive power of the model itself.

## Results

### Residue-residue contact prediction

The aim of the original DCA publication [8] was the identification of inter-protein residue-residue contacts in protein complexes, more precisely in the SK/RR complex in bacterial signal transduction. More recently, global methods for inferring direct co-evolution attacked the problem prediction of intra-domain contacts for large protein domain families [9]–[16], [26]. Thanks to the development of more efficient approximation techniques triggered by the wide availability of single-domain data on databases like Pfam [40], one can now easily undertake co-evolutionary analysis of a large number of protein families on normal desktop computer. To give a comparison, whereas the message-passing algorithm in [8] was limited to alignments with up to about 70 columns at a time (typically requiring some ad-hoc pre-processing of larger alignments to select the 70 potentially most interesting columns), the subsequent approaches easily handle MSA of proteins with up to ten times this number of columns.

In this context, our multivariate Gaussian DCA is particularly efficient: parameter estimation can be done explicitly in one step, and the computation of the relevant coupling measures such as the direct information (DI) and the log-likelihood also uses explicit analytical formulae. The analytical tractability of Gaussian probability distributions results in a major advantage in algorithmic complexity, and therefore in real running time. In the included implementation of the algorithm the largest alignment analyzed (PF00078, 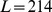 residues, 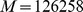 sequences) the DI is obtained in about 20 minutes, whereas a more typical alignment (e.g. PF00089, 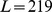, 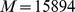) is analyzed in less than a minute on a normal 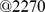 MHz Intel Core i5 M430 CPU on a Linux desktop. With respect to the computational complexity of the algorithm, the sequence reweighting step is 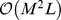 (since it requires a computation of sequence similarity for all sequence pairs in the MSA), while the model's parameters estimate is 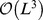 (since it requires to invert a covariance matrix whose size is proportional to 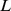).

**Figure 1:**
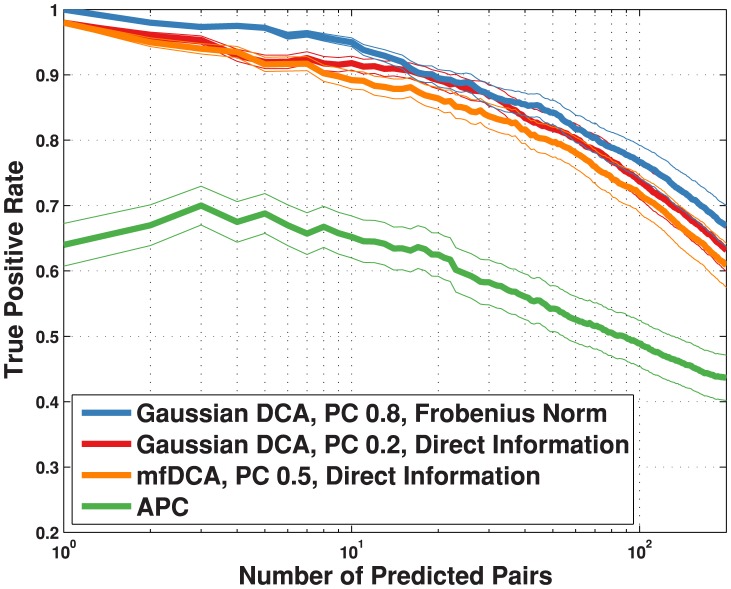
True positive rate plotted against number of predicted pairs. Results are shown for four different different scoring techniques: Frobenius norm (as described in [15], pseudo-count set to 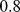, blue); Gaussian direct information (as described in the text, APC-corrected, pseudo-count set to 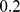, red); mean-field direct information (as described in [10], pseudo-count set to 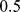, orange) and APC-corrected mutual information (as described in [41], green). The true positive rate is an arithmetic mean over 50 Pfam families (see Table 2 for the list); thin lines represent standard deviations.

Here, we will show that this gain in running time has no detectable cost in terms of predictive power. To this aim, we first studied the prediction of intra-domain contacts (see Fig. 1). From the Pfam database [40], a set of 50 families was selected for which the number of representative sequences is high enough to allow for a meaningful statistical analysis (average length 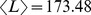 residues, average number of sequences per alignment 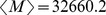), cf. the Methods section. For each family, 4 measures were determined: DI in mean-field approximation, DI and Frobenius norm (FN) in the Gaussian model, Average-product-corrected mutual information (MI) as described in [41]. As mentioned above, the FN in the Gaussian model is the same as that computed in the mean-field approximation of the discrete DCA model. Each measure was used to rank residue position pairs (only pairs which are at least 5 positions apart in the chain are considered), and high-ranking pairs are evaluated according to their spatial proximity in exemplary protein structures. A cutoff of 8 Å minimal distance between heavy atoms for contacts was chosen, in agreement with [10] and [42]. The best overall results are obtained with FN, as already noted in [15]; however, it is interesting to note that the Gaussian DI score is comparable to, and even slightly better then the mean-field DI score, which gives an important indication regarding the accuracy of the underlying probabilistic model: this in turn is relevant for subsequent analysis (see next section). Somewhat surprisingly, we also found that the optimal overall value of the pseudo-count parameter is strongly dependent on which scoring function is used: we explored the whole range 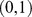 in steps of 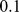, and found that the optimum for the FN score was at 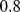, while for the DI score it was at 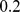.

**Figure 2:**
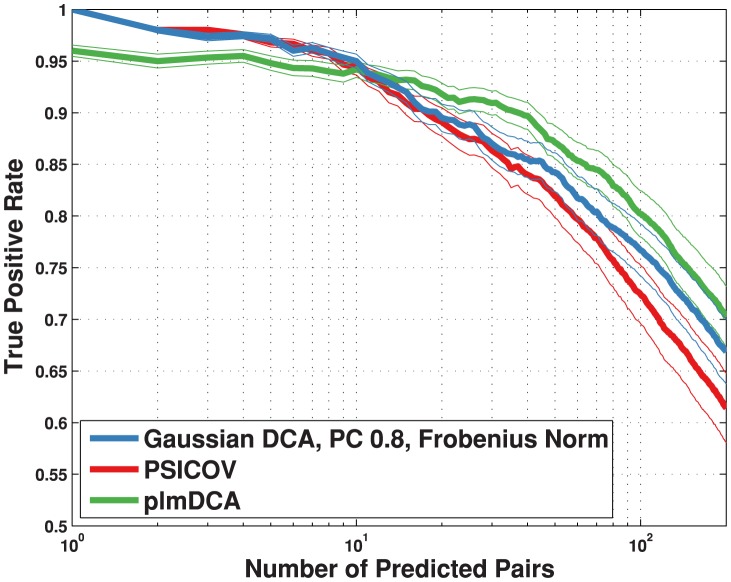
True positive rate plotted against number of predicted pairs. Data for plmDCA [15] (green) and PSICOV version 1.11 [12] (red) was obtained using the code provided by the authors with standard parameters as found in the distributed code, except that PSICOV was run with the -o flag to override the check against insufficient effective number of sequences. The true positive rate is an arithmetic mean over 50 Pfam families (see Table 2 for the list); thin lines represent standard deviations.

As a second test we ran on the same data-set a direct comparison between our method's best score, PSICOV [12] and plmDCA [15]. Fig. 2 shows that our method's performance is comparable to that of PSICOV (and even marginally better after the first 50 inferred couplings), and that the two methods are slightly better for the first 10 predicted contacts (with a 100% accuracy on the first contact). At ten predicted contacts, the true positive average is about 95% for all three methods. From ten predicted pairs on, both our method and PSICOV perform slightly worse than plmDCA: at 100 predicted contacts, the true positive rate is about 72% for PSICOV, 77% for the Gaussian model and 80% for plmDCA. A sample of running times for the three methods and different problem sizes, reported in Table 1, shows that our code can be at least an order of magnitude faster then PSICOV, and two orders of magnitude faster then plmDCA. These results suggest that our method is a good candidate for large scale problems of inference of protein contacts.

**Table 1:** Running times in seconds for a representative sample of proteins with varying length (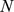) and sequences in alignment (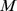), using different algorithms.

	PF00014	PF00025	PF00026	PF00078
N	53	175	317	214
M	4915	5460	4762	172360
Gaussian DCA (parallel)	0.7	5.3	16.3	534.8
Gaussian DCA (non-parallel)	1.7	12.7	52.1	3583.4
PSICOV	11.7	1141.9	5442.7	10965.1
plmDCA	433.2	6980.7	37364.8	303331.0

Since the Gaussian DCA code is parallelized, we show two series of results, one in which we used 8 cores and one in which we forced the code to run on a single core, for the sake of comparing with the non-parallel code of PSICOV and plmDCA. These benchmarks were taken on a 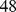-core cluster of 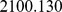 MHz AMD Opteron 6172 processors running Linux 3.5.0; PSICOV version 1.11 was used, compiled with gcc 4.7.2 at -O3 optimization level; plmDCA was run with MATLAB version r2011b. Gaussian DCA timings shown are taken using the Julia version of the code, using Julia version 0.2.

**Figure 3:**
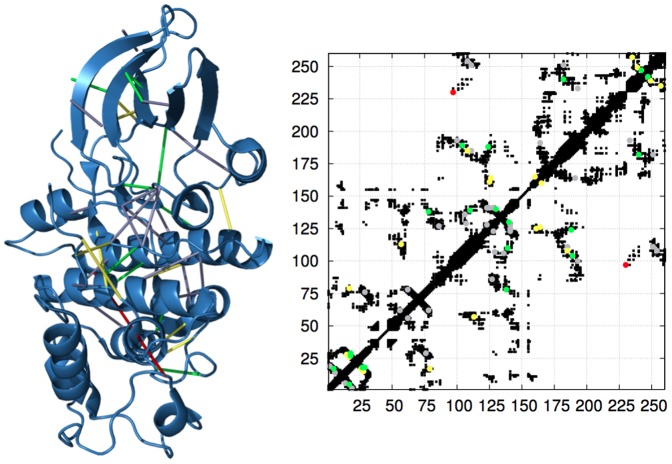
First 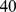 predicted contacts for the PF00069 family (Protein Kinase domain) with Gaussian DCA, using the same settings as for Fig. 2. The left panel shows the predicted contacts overlaid on the PDB structure *3fz1* (figure produced using the PyMOL software [51]); the right panel shows the predicted pairs overlaid on the contact map (true contacts as obtained by setting the threshold at 8 Å are shown in black). In both panels, the color code is the following: the first 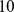 predicted contacts are depicted in green, the next 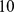 contacts in yellow, the last 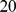 contacts in grey; the only false positive contact (occurring as the 24^th^ predicted pair) is shown in red.

Visual inspection of the predicted contacts does not reveal any significant bias with respect to the residue position, nor with respect to the sencondary or tertiary structures of the proteins. As an example, in Fig. 3 we show the first 40 predicted contacts (39 out of which are true positives) for the protein familiy PF00069 (Protein kinase domain) using the Gaussian DCA methods with the FN score: the pictures seem to indicate a sparse, fair sampling across the set of all true contacts.

**Figure 4:**
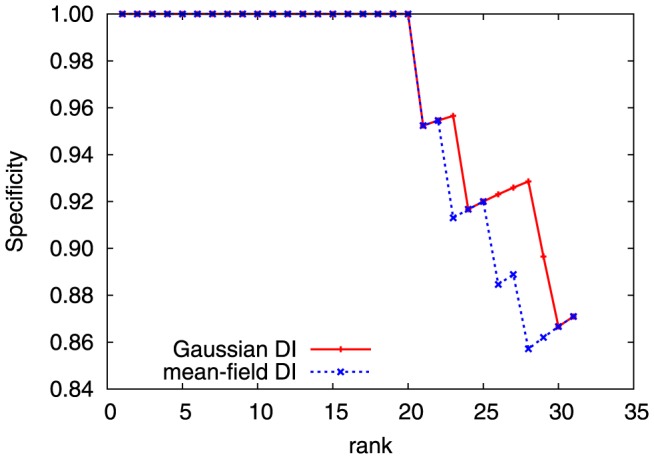
DI-ranking-induced mean true positive rate for predicting *inter-protein* contacts in the SK/RR complex, for both mean-field DCA (blue curve) and multivariate Gaussian DCA (red curve).

Finally, we have used the SK/RR data set containing 8,998 cognate SK/RR pairs, cf. Methods, to predict inter-protein residue-residue contacts. Results can be compared with those presented in [18], where the original message-passing DCA was applied to the same data-set, and 9 true contact prediction were reported before the first false positive appeared. In Fig. 4, results are shown for mean-field and Gaussian DCA, using the DI score: both methods improve substantially over the message-passing scheme (20 true positive predictions at specificity equal to one), but are highly comparable (with a little but not significant advantage of the Gaussian scheme). Again, we find that the improved efficiency and analytical tractability of Gaussian DCA comes at no cost for the predictive power.

### Predicting interactions between proteins in bacterial signal transduction

A typical bacterium uses, on average, about 20 two-component signal transduction systems to sense external signals, and to trigger a specific response. In bacteria living in complex environments, the number of different TCS may even reach 200. While the signals and consequently the mechanisms of signal detection vary strongly from one TCS to another, the internal phosphotransfer mechanism from the SK to the RR, which activates the RR, is widely conserved across bacteria: A majority of the kinase domains of SK belong to the protein domain family *HisKA* (PF00512), all RR to family *Response_reg* (PF00072) [40], cf. the Methods section. Despite their closely related functionality, the interactions in the different pathways have to be highly specific, to induce the correct specific answer for each recognized external signal.

A big fraction of SK and RR genes belonging to the same TCS pathway are co-localized in joint operons; the identification of the correct interaction partner is therefore trivial: such pairs are called cognate SK/RR. However, about 30% of all SK and 55% of all RR are so-called orphan proteins: their genes are isolated from potential interaction partners in the genome. While a large fraction of the RR are expected to be involved in other signal-transduction processes like chemotaxis, for each of the SK at least one target RR is expected to exist. It is a major challenge in systems biology to identify these partners, and to unveil the signaling networks acting in the bacteria. A step in this direction was taken in [17], [18], where co-evolutionary information extracted from cognate pairs is used to predict, with some success, orphan interaction partners.

An approach based on message-passing DCA [18] was tested in two well-studied model bacteria, namely *Caulobacter crescentus* (CC) and *Bacillus subtilis* (BS), where several orphan interactions are known experimentally [43]–[45]. The degree of accuracy of the method can be evinced from figure 4 of [18]: for CC, all known interactions between DivL, PleC, DivJ and CC_1062 with DivK and PleD are correctly reconstructed by the ranking obtained from the co-evolutionary scoring. Only in the case of the pair CenK-CenR, the signal is not sufficiently strong. For BS all the 5 orphan kinases KinA-B-C-D-E are known to interact with the RR Spo0F, which was clearly visible in co-evolutionary analysis in all but the KinB case.

**Figure 5:**
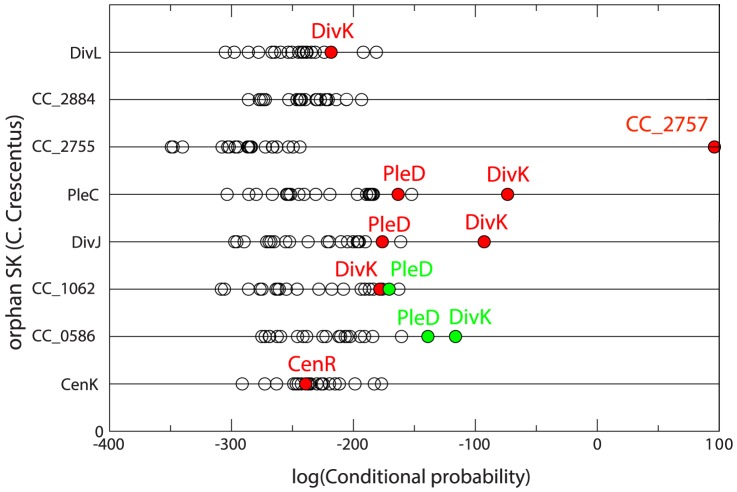
Partner prediction for *Caulobacter crescentus* orphan two-component proteins by the conditional probability method. Experimentally known interaction partners [44], [45] are shown in red. Green dots correspond to partner predictions suggested in [18]. As for [18], the overall performance of the algorithm is good, except for the prediction on CenK-CenR interaction.

**Figure 6:**
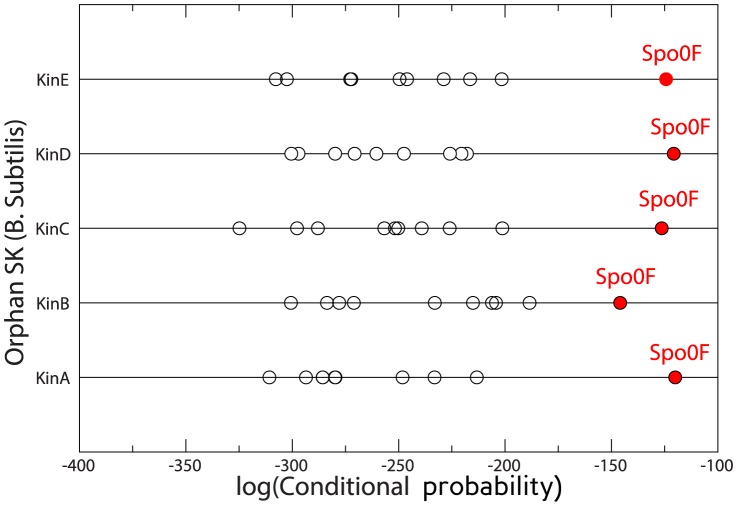
Partner prediction for *Bacillus subtilis* orphan two-component proteins. All 5 orphan kinases, KinA-E, are known to phosphorylate Spo0F, which is displayed in red and is always the maximally scoring protein in the RR set.

The method proposed here for orphans pairing relies on the Gaussian approximation and on the definition of the score 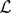, cf. Eq. 15 in Methods, which equals the log-odds ratio between the probabilities of two orphan sequences in the interacting model (inferred from cognate SK/RR alignments) and a non-interacting model (inferred independently from the two MSAs of the SK and the RR families). It is worth stressing at this point that all estimates of the likelihood score parameters are learned only on the cognates set. Ranked by 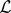, orphans interactions in CC are shown in Fig. 5. Results are very similar to those mentioned for [18]: known interactions are well reproduced for orphan kinases PleC and DivJ, while for CC_1062 and DivL the signal for an interaction with DivK, though present, is less clear. Finally, predictions for CC_0586 are identical in both studies but neither one is able to identify the CenK-CenR interaction. Fig. 6 shows predictions for orphan interactions in BS: observed interactions between KinA, KinB, KinC, KinD, KinE and Spo0F are manifest. This means that while predictions in CC are slightly less accurate compared to the message-passing strategy, predictions in BS show a greater accuracy.

## Discussion

In this work we have derived a multivariate Gaussian approach to co-evolutionary analysis, whereby we cast the problem of the inference of contacts in MSAs, as well as candidate interacting partners within two MSAs of interacting proteins, into a simple Bayesian formalism, under the hypothesis of normal inverse Wishart distribution of the Gaussian parameters.

The major advantage of this method is the very simple structure of the resulting probability distribution, which allows to derive analytical expressions for many relevant quantities (e.g. likelihoods and posterior probabilities). As a result, the computations performed with this model can be very efficient, as demonstrated by the code accompanying this paper.

Furthermore, our tests indicate that the prediction accuracy of residue contacts using the Gaussian model is comparable or superior to that achieved using the mean-field Potts model of [10], or by using the PSICOV method of [12] with default settings; accuracy in pairing interaction partners is comparable to that achieved in [18].

The simplicity and tractability of the model also suggests further directions for improvement. For example, the whole posterior distribution of relevant observables such as the DI could be studied and, possibly, used to provide more insight into the kind of predictions presented here (in particular, it could be used to measure the confidence on the predictions). Also, suitably designed, more informative priors (e.g. carrying biologically relevant information) could further enhance the prediction power of the method, although it is not obvious how to set a prior directly on the predicted interaction strengths, whereas with other methods – notably plmDCA [15] and PSICOV [12] – this should be straightforward. Finally, we observe that the log-likelihood score for interaction partners does not require an interaction model to be known in advance: the interaction partners can be identified across the whole families by optimizing the score of the joint alignment as a function of the mapping between potentially interacting partners, thus allowing to infer both the interacting elements and their inter-protein contacts at once.

## Materials and Methods

### Data

Input data is given as multiple sequence alignments of protein domains. For the first question (inference of residue-residue contacts in protein domains), we directly use MSAs downloaded from the Pfam database version 27.0 [40], [46], which are generated by aligning successively sequences to profile hidden Markov models (HMMs) [47] generated from curated seed alignments. We have selected 50 domain families, which were chosen according to the following criteria: (*i*) each family contains at least 2,000 sequences, to provide sufficient statistics for statistical inference; (*ii*) each family has at least one member sequence with an experimentally resolved high-resolution crystal structure available from the Protein Data Bank (PDB) [48], for assessing *a posteriori* the predictive quality of the purely sequence-based inference. The average sequence length of these 50 MSAs is 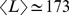 residues, the longest sequences are those of family PF00012 whose profile HMM contains 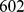 residues. The list of included protein domains, together with their PDB structure, is provided in Table 2.

**Table 2:** 50 Pfam families used in the benchmarks, together with their associated PDB entries.

Pfam ID	Description	PDB
PF00001	7 transmembrane receptor (rhodopsin family)	1f88, 2rh1
PF00004	ATPase family associated with various cellular activities (AAA)	2p65, 1d2n
PF00006	ATP synthase alpha/beta family, nucleotide-binding domain	2r9v
PF00009	Elongation factor Tu GTP binding domain	1skq, 1xb2
PF00011	Hsp20/alpha crystallin family	2bol
PF00012	Hsp70 protein	2qxl
PF00013	KH domain	1wvn
PF00014	Kunitz/Bovine pancreatic trypsin inhibitor domain	5pti
PF00016	Ribulose bisphosphate carboxylase large chain, catalytic domain	1svd
PF00017	SH2 domain	1o47
PF00018	SH3 domain	2hda, 1shg
PF00025	ADP-ribosylation factor family	1fzq
PF00026	Eukaryotic aspartyl protease	3er5
PF00027	Cyclic nucleotide-binding domain	3fhi
PF00028	Cadherin domain	2o72
PF00032	Cytochrome b(C-terminal)/b6/petD	1zrt
PF00035	Double-stranded RNA binding motif	1o0w
PF00041	Fibronectin type III domain	1bqu
PF00042	Globin	1cp0
PF00043	Glutathione S-transferase, C-terminal domain	6gsu
PF00044	Glyceraldehyde 3-phosphate dehydrogenase, NAD binding domain	1crw
PF00046	Homeobox domain	2vi6
PF00056	Lactate/malate dehydrogenase, NAD binding domain	1a5z
PF00059	Lectin C-type domain	1lit
PF00064	Neuraminidase	1a4g
PF00069	Protein kinase domain	3fz1
PF00071	Ras family	5p21
PF00072	Response regulator receiver domain	1nxw
PF00073	Picornavirus capsid protein	2r06
PF00075	RNase H	1f21
PF00077	Retroviral aspartyl protease	1a94
PF00078	Reverse transcriptase (RNA-dependent DNA polymerase)	1dlo
PF00079	Serpin (serine protease inhibitor)	1lj5
PF00081	Iron/manganese superoxide dismutases, alpha-hairpin domain	3bfr
PF00082	Subtilase family	1p7v
PF00084	Sushi domain (SCR repeat)	1elv
PF00085	Thioredoxin	3gnj
PF00089	Trypsin	3tgi
PF00091	Tubulin/FtsZ family, GTPase domain	2r75
PF00092	Von Willebrand factor type A domain	1atz
PF00102	Protein-tyrosine phosphatase	1pty
PF00104	Ligand-binding domain of nuclear hormone receptor	1a28
PF00105	Zinc finger, C4 type (two domains)	1gdc
PF00106	Short chain dehydrogenase	1a27
PF00107	Zinc-binding dehydrogenase	1a71
PF00108	Thiolase, N-terminal domain	3goa
PF00109	Beta-ketoacyl synthase, N-terminal domain	1ox0
PF00111	2Fe-2S iron-sulfur cluster binding domain	1a70
PF00112	Papain family cysteine protease	1o0e
PF00113	Enolase, C-terminal TIM barrel domain	2al2

Following [12], we discarded the sequences in which the fraction of gaps was larger then 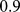. However, in [12], an additional pre-processing stage was applied, in which a target sequence is chosen as the one for which prediction of contacts is desired, and all residue positions in the alignment (i.e. columns in the alignment matrix 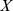) where the target sequence alignment has gaps are removed. We did not find this pre-processing step to improve the prediction, for either PSICOV or our model, and therefore all results presented in this work do not include this additional filtering.

For the second question (identification of interaction partners), we have used the data of [18], thus having the possibility to directly compare with previous results. In summary (for details see [18]), this data comes from 769 bacterial genomes, scanned using HMMER2 with the Pfam 22.0 HMMs for the Sensor Kinase (SK) domain *HisKA* (PF00512) and for the Response Regulator domain *Response_reg* (PF00072) [49], resulting in 12,814 SK and 20,368 RR sequences.

A total of 8,998 SK-RR pairs are found to be cognates, i.e. to be coded by genes in common operons, while the rest are so-called orphans. For statistical inference, cognates sequences are concatenated into a single MSA, each line containing exactly one SK and its cognate RR.

### A binary representation of MSA

The data we use are MSAs for large protein-domain families. An MSA provides a 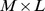-dimensional array 
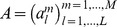
: each row contains one of the 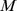 aligned homologous protein sequences of length 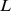. Sequence alignments are formed by the 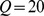 different amino-acids, and may contain alignment gaps, and therefore the total alphabet size is 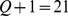. For simplicity, we denote amino-acids by numbers 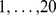, and the gap by 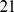.

**Figure 7:**
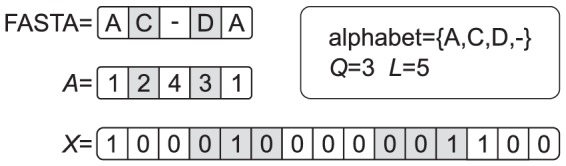
Illustration of the encoding of a sequence from FASTA format to its intermediate numeric representation (matrix 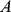) to its final binarized representation (matrix 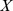). For clarity, we restrict the alphabet to 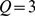 amino-acids, 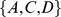, plus the gap. The alternation of white and gray cell backgrounds helps to track the transformation (e.g. 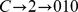). Typically, MSAs of protein families are such that in every column (i.e. residue position) there appears a number of distinct residues smaller than or equal to 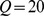. Here, we did not not consider a restriction of the alphabet to the residues actually occurring, and we used instead the same encoding for all residues.

Here we consider a modified representation, similar to that used in [12], which turns out to be more practical for the multivariate modeling we are going to propose (cf. Fig. 7). The MSA is transformed into a 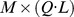-dimensional array 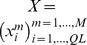 over a binary alphabet 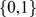. More precisely, each residue position in the original alignment is mapped to 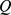 binary variables, each one associated with one standard amino-acid, taking value one if the amino-acid is present in the alignment, and zero if it is absent; the gap is represented by 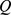 zeros (i.e. no amino-acid is present). Consequently, at most one of the 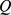 variables can be one for a given residue position. For each sequence, the new variables are collected in one row vector, i.e. 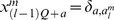 for 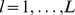 and 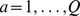. The Kronecker symbol 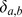 equals one for 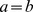, and zero otherwise.

Denoting the row length of 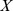 as 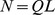, we introduce its empirical mean 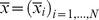 and the empirical covariance matrix 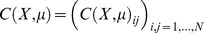 for given mean 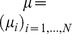:
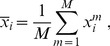
(1)

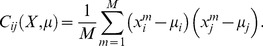
(2)


The empirical covariance is thus 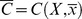. Note that the entry 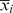, with 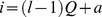, measures the fraction of proteins having amino-acid 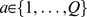 at position 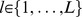. Similarly, the entry 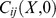 of the correlation matrix, with 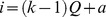 and 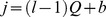, is the fraction of proteins which show simultaneously amino-acid 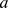 in position 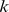 and 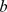 in position 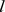.

#### The Gaussian model

We develop our multivariate Gaussian approach by approximating the binary variables as real-valued variables. Even though the former are highly structured, due to the fact that at most one amino-acid is present in each position of each sequence, we will not enforce these constraints on the model. Instead, we shall rely on the fact that the constraint is present by construction in the input data, and that as a consequence we have, for any residue position 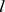 and any two states 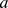 and 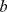 with 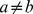:
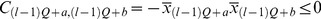
(3)i.e. two different amino-acids at the same site are anti-correlated. Therefore, we shall let the parameter inference machinery work out suitable couplings between different amino-acid values at the same site, which generate these observed anti-correlations.

The multivariate Gaussian model and the Bayesian inference of its parameters are well-studied subjects in statistics, thus here we only briefly review the main ideas behind our approach, referring to [50] for details. The multivariate Gaussian distribution is parametrized by a mean vector 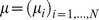 and a covariance matrix 
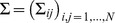
. Its probability density is
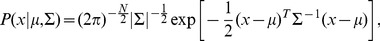
(4)



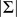
 being the determinant of 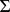, and it turns out that the 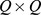 block
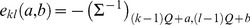
(5)(with 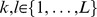 and 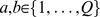) plays the role of the direct interaction term in DCA between residues 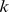 and 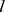. Assuming for the moment statistical independence of the 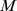 different protein sequences in the MSA, the probability of the data 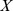 under the model (i.e. the likelihood) reads
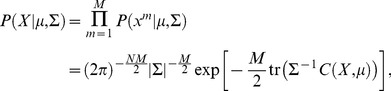
(6)with 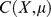 given by Eq. 2.

When the empirical covariance 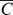 is full rank, the likelihood attains its maximum at 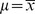 and 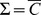, which constitute the parameter estimates within the maximum likelihood approach. However, due to the under-sampling of the sequence space, 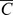 is typically rank deficient and this inference method is unfeasible. To estimate proper parameters, we make use of a Bayesian inference method, which needs the introduction of a prior distribution over 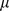 and 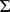. The required estimate is then computed as the mean of the resulting posterior, which is the parameter distribution conditioned to the data. As we have already mentioned, a convenient prior is the conjugate prior, which gives a posterior with the same structure as the prior but identified by different parameters accounting for the data contribution. The conjugate prior of the multivariate Gaussian distribution is the normal-inverse-Wishart (NIW) distribution. A NIW prior has the form 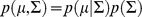, where
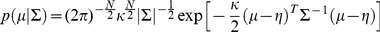
(7)is a multivariate Gaussian distribution on 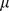 with covariance matrix 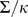 and prior mean 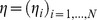. The parameter 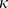 has the meaning of number of prior measurements. The prior on 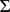 is the inverse-Wishart distribution
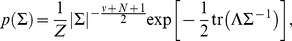
(8)where 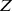 is a normalizing constant:
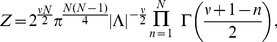
(9)

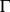
 being Euler's Gamma function. The parameters 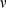 and 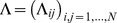 are the degree of freedom and the scale matrix, respectively, shaping the inverse-Wishart distribution. The condition for this distribution to be integrable is 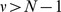. The posterior 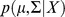, proportional to 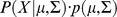, is still a NIW distribution, as one can easily verify starting from Eqs. 6, 7 and 8. The posterior distribution 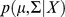 is characterized by parameters 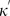, 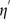, 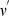, and 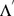 given by the formulae
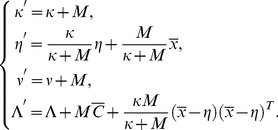
(10)


The mean values of 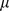 and 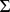 under the NIW prior are 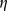 and 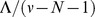, and, similarly, their expected values under the NIW posterior are 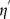 and 
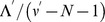
, respectively. Our estimates of the mean vector and the covariance matrix, that with a slight abuse of notation we shall still denote by 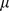 and 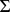 for the sake of simplicity, are thus
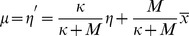
(11)and
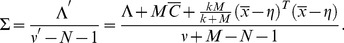
(12)


The NIW posterior is maximum at 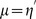 and 
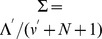
, with the consequence that the *maximum a posteriori* estimate would provide the same estimate of 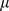 and an estimate of 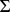 that only differs from the previous one by a scale factor.

As a first attempt of protein contact prediction by means of the present model, we choose 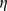 and 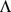 to be as uninformative as possible. In particular, since 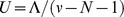 is the prior estimate of 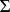, it is natural to set 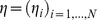 and 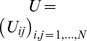 to the mean and the covariance matrix of uniformly distributed samples. Therefore, we set 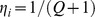 for any 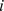, and 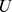 to a block-matrix composed of 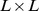 blocks of size 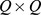 each, where the out-of-diagonal blocks are uniformly 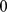:
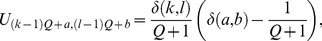
(13)where 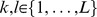 and 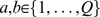, and 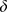 is the Kronecker's symbol. Moreover, we choose 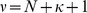 in order to reconcile Eq. 12 with the pseudo-count-corrected covariance matrix of [10] with pseudo-count parameter 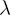. Indeed, identifying 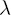 with 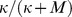, this instance allows us to recast the estimation of 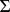 as
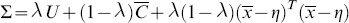
(14)and 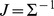 becomes the same as in the mean-field Potts model. Manifestly from here, the effect of the prior is enhanced by values of 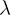 close to 1 while it is negligible when 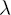 approaches 0. Interestingly, the Gaussian framework provides an interpretation of the pseudo-count correction in terms of a prior distribution, which may allow improving the inference issue by exploiting more informative prior choices.

#### Reweighted frequency counts

The approach outlined in the above sections assumes that the rows of the MSA matrix 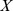, i.e. the different protein sequences, form an independently and identically distributed (i.i.d.) sample, drawn from the model distribution, cf. Eq. 6. For biological sequence data this is not true: there are strong sampling biases due to phylogenetic relations between species, due to the sequencing of different strains of the same species, and due to a non-random selection of sequenced species. The sampling is therefore clustered in sequence space, thereby introducing spurious non-functional correlations, whereas other viable parts of sequence space (in the sense of sequences which would fall into the same protein family) are statistically underrepresented. To partially remove this sampling bias, we use the same re-weighting scheme used in the PSICOV version 1.11 code [12] (which is the same as that used in [8], [10], with an additional pre-processing pass to estimate a value for the similarity threshold; see File S1 for details). The procedure can be seen as generalization of the elimination of repeated sequences.

#### Computing the ranking score

Contact prediction using DCA relies on ranking pairs of residue positions 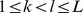 according to their direct interaction strength. As mentioned before, two positions interact via a 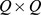 matrix 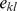 given by Eq. 5. To compare two position pairs 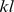 and 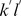, we need to map these matrices to a single scalar quantity. We have tested two different transformations: the first one, following [8], is the so-called direct information (DI), which measures the mutual information induced only by the direct coupling 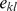 between two positions 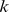 and 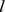 (for a more precise definition see File S1); the second one, following [15], is the Frobenius norm (FN) of the sub-matrix obtained by (*i*) changing the gauge of the interaction such that the sum of each row and column is zero, and (*ii*) removing the row and column corresponding to the gap symbol. In our empirical tests (cf. Fig. 1), the FN score can reach a better overall accuracy in residues contacts prediction; the DI score, however, also achieves good results, is gauge-invariant, and has a clear interpretation in terms of the underlying model: it is therefore a useful indicator to compare the Gaussian model with the mean-field approximation to the discrete model. In the multivariate Gaussian setting, the DI can be calculated explicitly, as shown in File S1, thus resulting in a gain in computation time as compared to the mean-field DCA in [10], while achieving similar or better performance (cf. Fig. 1).

We found empirically that both the DI and the FN scores produce slightly better results in the residue contact prediction tests when adjusted via average-product-correction (APC), as described in [41].

### Summary of the residue contact prediction steps

To summarize the previous sections, here we list the steps which are taken in order to get from a MSA to the contact prediction:

- clean the MSA by removing inserts and keeping only matched amino acids and deletions;
- remove the sequences for which 90% or more of the entries are gaps;
- assign a weight to each sequence, and compute the reweighted frequency counts 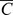 and 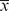 (see Eqs. 1 and 2, and Suporting File S1);
- estimate the correlation matrix 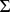 by means of Eq. 14;
- compute 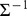, and divide it in 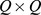 blocks 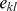 (see Eq. 5);
- for each pair 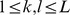, compute a score (DI or FN) from 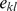, thus obtaining an 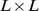 symmetric matrix 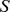 (with zero diagonal);
- apply APC to the score matrix (i.e. subtract to each entry 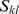 the product of the average score over 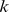 and the average score over 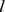, divided by the overall score average – the averages are computed excluding the diagonal), and obtain an adjusted score matrix 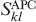;
- rank all pairs 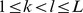, with 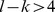, in descending order according to 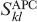.

### A log-likelihood score for protein-protein interaction

In [18], DCA has been used to predict RR interaction partners for orphan SK proteins in bacterial TCS, and to detect crosstalk between different cognate SK/RR pairs. Relying on the improved efficiency of the multivariate Gaussian approach presented here, we can introduce a much clearer but similarly performing definition of a protein-protein interaction score.

This score is based on the existence of a large set of known interaction partners: we collect them in a unified MSA, in which each row contains the concatenation of two interacting protein sequences, and we encode them in a matrix denoted by 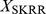. The encoded MSAs restricted to each of the single protein families are denoted by 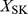 and 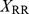. We estimate model parameters 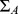 and 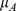 for each of the three alignments 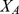, with 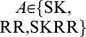. Whereas the parameters for the two alignments of single protein families describe the *intra*-domain co-evolution inside each domain, the parameter matrix 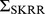, obtained from the joint MSA, also models the *inter*-protein co-evolution.

In order to decide if two new sequences 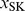 and 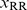 interact, we first introduce the sequence 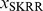 as the (horizontal) concatenation of 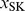 with 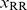. Next we define a log-odds ratio comparing the probability of these sequences under the joint SKRR-model with the one under the separate models for SK and RR, i.e. we calculate
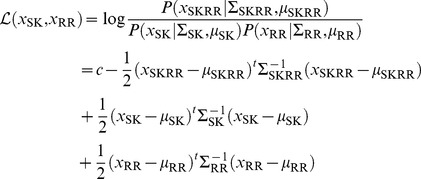
(15)


with 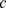 being a constant (i.e. not depending on the sequence 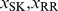) coming from the normalization of the multivariate Gaussians. Intuitively, this score measures to what extent the two sequences are coherent with the model of interacting SK/RR sequences, as compared to a model which assumes them to be just two arbitrary (and thus typically not interacting) SK and RR sequences. In mathematical terms, it can also be seen as the log-odds ratio between the conditional probability of 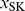 knowing 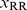, and the unconditioned probability of 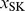.

## Supporting Information

File S1 (PDF)
