# Supplementary material for: Fast and Accurate Multivariate Gaussian Modeling of Protein Families: Predicting Residue Contacts and Protein-Interaction Partners
Source: PLoS One. 2014 Mar 24;9(3):e92721. doi: 10.1371/journal.pone.0092721 (PMC3963956; doi:10.1371/journal.pone.0092721)
Supplement: File S1 — (PDF) [file pone.0092721.s001.pdf]

Fast and accurate multivariate Gaussian modeling of protein families: Predicting residue contacts and protein-interaction partners – Supplementary Matrials

Carlo Baldassi[1,2,†], Marco Zamparo[1,2,†], Christoph Feinauer[1], Andrea Procaccini[2], Riccardo Zecchina[1,2], Martin Weigt[3,4], Andrea Pagnani[1,2,*]
[1] DISAT and Center for Computational Sciences, Politecnico di Torino, Corso Duca degli Abruzzi 24, I-10129 Torino, Italy
[2]Human Genetics Foundation-Torino, Molecular Biotechnology Center, Via Nizza 52, I-10126 Torino, Italy
[3] Sorbonne Universités, UPMC Univ Paris 06, UMR 7238, Computational and Quantitative Biology, 15 rue de l'Ecole de Médecine, 75006 Paris, France
[4] CNRS, UMR 7238, Computational and Quantitative Biology, 15 rue de l'Ecole de Médecine, 75006 Paris, France
∗ E-mail: andrea.pagnani@polito.it
† These authors contributed equally to this work

1 Direct Information computation in the Gaussian model

In order to implement DCA, we aim at quantifying the effect of the interaction between each pair of residues. The idea is to compare a system with only two interacting residues with the non–interacting corresponding scene. Single–site marginals are preserved in both cases while the interaction term is encoded in the matrix $J = \Sigma^{-1}$ as derived in the Main Text. Indeed, the interaction between residues l and l' is described by $e_{ll'} := -\hat{J}_{ll'}$, denoting with $\hat{J}_{ll'}$ the $Q \times Q$ block of J corresponding to residues l and l', which is the $Q \times Q$ matrix with entries $(J)_{nn'}$ such that $n = l \mod Q$ and $n' = l' \mod Q$.

The non–interacting case is easily approached by repeating the Bayesian analysis described in the Main Text independently for each residue l and provides $P_l^{\text{ind}}(x)$, where now x is the Q-state vector associated to position l. As a result, P_l^{ind} is a Gaussian distribution with the blocks corresponding to l of μ and Σ, given in the Main Text respectively, as mean and covariance. The interacting instance between l and l' is instead characterized by the Gaussian distribution $P_{ll'}^{\text{dir}}(x, x')$ with interaction $\hat{J}_{ll'}$ and single–site marginals $P_l^{\text{ind}}(x)$ and $P_{l'}^{\text{ind}}(x')$. Notice that $P_{ll'}^{\text{dir}}(x, x')$ reduces to $P_l^{\text{ind}} \otimes P_{l'}^{\text{ind}}(x, x') := P_l^{\text{ind}}(x) P_{l'}^{\text{ind}}(x')$ when $\hat{J}_{ll'} = 0$. In order to measure the strength of $\hat{J}_{ll'}$ we then define the direct information $DI_{ll'}$ between sites l and l' as the Kullback–Leibler divergence between $P_{ll'}^{\text{dir}}$ and $P_l^{\text{ind}} \otimes P_{l'}^{\text{ind}}$:

$$DI_{ll'} := KL\left(P_{ll'}^{\text{dir}} || P_l^{\text{ind}} \otimes P_{l'}^{\text{ind}}\right). \tag{1}$$

We have $DI_{ll'} \geq 0$ and $DI_{ll'} = 0$ if $\hat{J}_{ll'} = 0$.

We stress that the expression of the direct information is gauge–invariant, in the sense that it is independent of the a.a. index omitted in the model construction. Moreover, even though the matrix J is the same as in the mean–field approximation of the Potts model [1,2], $DI_{ll'}$ is different.

Here we show how to compute the direct information $DI_{ll'}$ between residues l and l'. We denote with $\hat{\mu}_l$ the mean associated to position l, which is the Q-vector with entries $(\mu)_n$ such that $r(n) = l$. Similarly to $\hat{J}_{ll'}$, $\hat{\Sigma}_{ll'}$ represent the $Q \times Q$ block of Σ with entries $(\Sigma)_{nn'}$ where $r(n) = l$ and $r(n') = l'$. The Gaussian distribution P_l^{ind} is characterized by mean $\hat{\mu}_l$ and covariance $\hat{\Sigma}_{ll}$. The Gaussian distribution $P_{ll'}^{\text{dir}}$ has marginals P_l^{ind} and $P_{l'}^{\text{ind}}$ and retains $\hat{J}_{ll'}$ to describe the interaction between the two residues. Thus, writing its mean as (α, β) with $\alpha, \beta \in \mathbb{R}^Q$ and its interaction (or precision) matrix as

$$\begin{pmatrix} H & \hat{J}_{ll'} \\ \hat{J}_{l'l} & K \end{pmatrix} \tag{2}$$

with $H, K \in \mathbb{R}^{Q\times Q}$ symmetric positive definite, the marginalization constraints impose that $\alpha = \hat{\mu}_l$ and $\beta = \hat{\mu}_{l'}$ and that the diagonal block of

$$\begin{pmatrix} H & \hat{J}_{ll'} \\ \hat{J}_{l'l} & K \end{pmatrix}^{-1} \tag{3}$$

equal $\hat{\Sigma}_{ll}$ and $\hat{\Sigma}_{l'l'}$ respectively. Exploiting the formula for the block-wise inversion of block matrices, the conditions on H and K can be explicitly stated as

$$\begin{cases} H - \hat{J}_{ll'}K^{-1}\hat{J}_{l'l} = \hat{\Sigma}_{ll}^{-1}; \\ K - \hat{J}_{l'l}H^{-1}\hat{J}_{ll'} = \hat{\Sigma}_{l'l'}^{-1}. \end{cases} \tag{4}$$

The direct information $DI_{ll'}$ is the Kullback–Leibler divergence

$$DI_{ll'} = KL\left(P_{ll'}^{\mathrm{dir}}||P_l^{\mathrm{ind}} \otimes P_{l'}^{\mathrm{ind}}\right) := \int_{\mathbb{R}^Q} dx \int_{\mathbb{R}^Q} dx' \ln\left(\frac{P_{ll'}^{\mathrm{dir}}(x,x')}{P_l^{\mathrm{ind}}(x)\, P_{l'}^{\mathrm{ind}}(x')}\right) P_{ll'}^{\mathrm{dir}}(x,x')\,. \tag{5}$$

Simple algebra shows that

$$DI_{ll'} = \frac{1}{2}\left[\ln\det\begin{pmatrix} H & \hat{J}_{ll'} \\ \hat{J}_{l'l} & K \end{pmatrix} + \ln\det\hat{\Sigma}_{ll} + \ln\det\hat{\Sigma}_{l'l'}\right]. \tag{6}$$

Recalling that $\hat{\Sigma}_{ll}$ is a symmetric positive definite matrix for any l is of some help in order to explicit $DI_{ll'}$. Indeed, this fact tells us that $\hat{\Sigma}_{ll}$ admits the Cholesky decomposition $\hat{\Sigma}_{ll} = S_l S_l^T$ with invertible Cholesky factor S_l. Let us then introduce the matrices $T_{ll'} := S_l^T \hat{J}_{ll'} S_{l'}$, $X := S_l^T H S_l$ and $Y := S_{l'}^T K S_{l'}$. We have that $T_{ll'}^T = T_{l'l}$ as a consequence of the relation $\hat{J}_{l'l} = \hat{J}_{ll'}^T$ due to the symmetry of J. With these definitions we can recast $DI_{ll'}$ as

$$\begin{aligned} 2DI_{ll'} &= \ln\det\begin{pmatrix} H & \hat{J}_{ll'} \\ \hat{J}_{l'l} & K \end{pmatrix} + \ln\det\begin{pmatrix} S_l S_l^T & 0 \\ 0 & S_{l'}S_{l'}^T \end{pmatrix} \\ &= \ln\det\begin{pmatrix} H & \hat{J}_{ll'} \\ \hat{J}_{l'l} & K \end{pmatrix} + \ln\det\begin{pmatrix} S_l & 0 \\ 0 & S_{l'} \end{pmatrix} + \ln\det\begin{pmatrix} S_l^T & 0 \\ 0 & S_{l'}^T \end{pmatrix} \\ &= \ln\det\begin{pmatrix} S_l^T & 0 \\ 0 & S_{l'}^T \end{pmatrix}\cdot\begin{pmatrix} H & \hat{J}_{ll'} \\ \hat{J}_{l'l} & K \end{pmatrix}\cdot\begin{pmatrix} S_l^T & 0 \\ 0 & S_{l'}^T \end{pmatrix} = \ln\det\begin{pmatrix} X & T_{ll'} \\ T_{ll'}^T & Y \end{pmatrix}. \end{aligned} \tag{7}$$

In addition, starting from eq. 4, we can write down corresponding equations for X and Y:

$$\begin{cases} X = I + T_{ll'}Y^{-1}T_{ll'}^T; \\ Y = I + T_{ll'}^T X^{-1} T_{ll'}, \end{cases} \tag{8}$$

being I the identity $Q\times Q$-matrix. Notice that X and Y must constitute the positive definite solution of this problem. Interestingly, the latter of these equations gives

$$\begin{pmatrix} X & T_{ll'} \\ T_{ll'}^T & Y \end{pmatrix} = \begin{pmatrix} X & 0 \\ T_{ll'}^T & I \end{pmatrix}\cdot\begin{pmatrix} I & X^{-1}T_{ll'} \\ 0 & I \end{pmatrix}. \tag{9}$$

Then, the property of block matrices

$$\det\begin{pmatrix} A & C \\ 0 & B \end{pmatrix} = \det\begin{pmatrix} A & 0 \\ C & B \end{pmatrix} = \det A \det B \tag{10}$$

provides the formula

$$DI_{ll'} = \frac{1}{2} \ln \det X. \tag{11}$$

As far as the solution of eq. 8 is concerned, let us observe that $X^{-1}T_{ll'} = T_{ll'}Y^{-1}$, as one recognizes multiplying the first equation by $X^{-1}T_{ll'}$ on the left and the second one by $T_{ll'}Y^{-1}$ on the right. The consequence of this identity is that the matrix X satisfies the relation $X^2 - X - T_{ll'}T_{ll'}^T = 0$, which is equivalent to the first of eq. 8 after the substitution of $T_{ll'}Y^{-1}$ with $X^{-1}T_{ll'}$. The matrix $T_{ll'}T_{ll'}^T$ is symmetric positive semi-definite and denoting with $t_{ll'}^1 \le t_{ll'}^2 \le \cdots \le t_{ll'}^Q$ its eigenvalues, not necessarily distinct, we have that X has eigenvalues

$$\frac{1+\sqrt{1+4t_{ll'}^1}}{2} \le \frac{1+\sqrt{1+4t_{ll'}^2}}{2} \le \cdots \le \frac{1+\sqrt{1+4t_{ll'}^Q}}{2}. \tag{12}$$

The fact that X is positive definite has been exploited here for determining its spectrum. As the final result we get

$$DI_{ll'} = \frac{1}{2}\sum_{q=1}^{Q} \ln\left(\frac{1+\sqrt{1+4t_{ll'}^q}}{2}\right). \tag{13}$$

2 Reweighting scheme

We used the same reweighting scheme used in the PSICOV version 1.11 code [3], to compensate for the sampling bias introduced by phylogenetic relations between species. We report the details of the computations here for convenience.

Weights are computed in two steps: a pre-processing step which is used to compute a similarity threshold r, and a weight-computation step which is the same as that used in [4] and uses r as a parameter.

The similarity threshold r is defined as being inversely proportional to the average sequence identity, i.e. the average, over all pairs of sequences, of the fraction of identical amino-acids in corresponding residues of two sequences. The constant of proportionality is chosen as $0.32 \cdot 0.38 = 0.1216$, which gives good overall results. As a further refinement, r is clamped such that its value cannot exceed 0.5.

The threshold r is then used to define neighborhoods around each sequence: only sequences with less than rL identical amino-acids are considered to carry independent information, and so for each protein sequence $a^m = (a_1^m, \ldots, a_L^m)$, $m = 1, \ldots, M$, in the MSA we count the number n^m of sequences with at least rL identical amino-acids (including a^m itself into this count), and we re-weight the influence of the sequence by the factor $w^m = 1/n^m$. This leads to a redefinition of the empirical means and covariances (see eqs. 1 and 2 in the Main Text), for $1 \le i, j, \le N$:

$$\bar{x}_i = \frac{1}{M_{\text{eff}}} \sum_{m=1}^{M} w^m x_i^m \tag{14}$$

$$\bar{C}_{ij} = \frac{1}{M_{\text{eff}}} \sum_{m=1}^{M} w^m \left(x_i^m - \bar{x}_i\right)\left(x_j^m - \bar{x}_j\right), \tag{15}$$

where $M_{\text{eff}} = \sum_{m=1}^{M} w^m$ is a normalization factor, which can be understood as the effective number of independent sequences. These re-weighted empirical means are used for estimating the model parameters (see eqs. 11 and 12 in the Main Text).

References

1. Morcos F, Pagnani A, Lunt B, Bertolino A, Marks DS, et al. (2011) Direct-coupling analysis of residue coevolution captures native contacts across many protein families. Proceedings of the National Academy of Sciences 108: E1293-E1301.

2. Marks DS, Colwell LJ, Sheridan R, Hopf TA, Pagnani A, et al. (2011) Protein 3d structure computed from evolutionary sequence variation. PLoS ONE 6: e28766.

3. Jones DT, Buchan DWA, Cozzetto D, Pontil M (2012) PSICOV: precise structural contact prediction using sparse inverse covariance estimation on large multiple sequence alignments. Bioinformatics 28: 184.

4. Weigt M, White RA, Szurmant H, Hoch JA, Hwa T (2009) Identification of direct residue contacts in protein-protein interaction by message passing. Proceedings of the National Academy of Sciences 106: 67-72.
